# Mental Toughness and 2K Rowing Performance in Division II Female Collegiate Athletes: A Longitudinal Analysis Using Mixed-Effects Modeling

**DOI:** 10.3390/sports14070282

**Published:** 2026-07-06

**Authors:** Zacharias Papadakis, Andreas Stamatis

**Affiliations:** 1 Department of Health Sciences & Clinical Practice, Barry University, Miami Shores, FL 33161, USA; 2 Department of Health & Sport Sciences, University of Louisville, Louisville, KY 40292, USA; andreas.stamatis@louisville.edu; 3 Sports Medicine Institute, University of Louisville Health, Louisville, KY 40208, USA

**Keywords:** mental toughness, rowers, 2K time trial, collegiate athletes, repeated measures

## Abstract

Mental toughness (MT) may contribute to within-athlete rowing performance variation, yet longitudinal evidence remains sparse. This pilot study examined within-athlete associations between MT and 2K ergometer performance across a competitive season in Division II female rowers. Twelve athletes (age 20.8 ± 2.1 years) completed the mental toughness index (MTI) before four standardized 2K time trials. Performance was modeled using a linear mixed-effects model with a random intercept for the athlete. The MTI was decomposed into within- and between-athlete components, with the timepoint as a categorical covariate. Small-sample inference used CR2 cluster-robust standard errors with Satterthwaite degrees of freedom. Performance improved mid-season relative to baseline (Timepoint 3: −7.29 s; 95% CI [−13.29, −1.29]; *p* = 0.023). The within-athlete MTI association was small and imprecise (*β* = −0.48 s/point; 95% CI [−1.56, 0.59]; *p* = 0.311), and the between-athlete MTI was unassociated with performance (*β* = 0.70; *p* = 0.667). Stable between-athlete differences dominated over variability (ICC = 0.946; *R*^2^_m_ = 0.033; *R*^2^_c_ = 0.948). The within-athlete MTI estimate was small and imprecise; given the wide compatibility interval, both the direction and magnitude of the association remain highly uncertain, and this inconclusive finding should not be interpreted as evidence of absence. Future studies with larger samples and key covariates (e.g., training load and illness/injury) are needed to confirm these preliminary estimates.

## 1. Introduction

Competitive sports are inherently characterized by the relentless pursuit of factors that enhance athletic performance, ranging from physiological and biomechanical aids to psychological strategies [1]. As athletes and coaches seek to leverage every potential advantage, psychological strategies have emerged as possible predictors of athletic performance [2,3,4], with both US (e.g., NCAA) and global initiatives (e.g., International Olympic Committee) focusing on athletes’ holistic development and psychological well-being [5,6].

Among these, mental toughness (MT) has emerged as a critical psychological attribute, and it is often cited as a key differentiator among athletes possessing similar levels of athletic performance (e.g., choking) [7,8,9]. In this study, we draw on Gucciardi’s contemporary conceptualization of MT as a state-like psychological resource that is purposeful, flexible, and efficient for goal-directed pursuits [10], and we operationalize MT using the eight-item mental toughness index developed and validated by Gucciardi et al. [11]. Unlike early definitions that viewed MT as a fixed personality trait, this framework posits that MT is a dynamic resource that can fluctuate across a season in response to stress, adversity, and fatigue that can be evaluated via the eight-item mental toughness index (MTI) [11,12]. This distinction is important for high-performance contexts because it frames MT not simply as something athletes possess, but as a psychological resource that may vary across demanding training and competition periods.

Despite advances in conceptualizing MT and this conceptual clarity, contemporary sport psychology scholarship has raised important questions about the discriminant validity of MT relative to adjacent constructs. Substantial overlap has been documented between MT, grit, resilience, self-efficacy, and psychological flexibility [13,14,15,16,17,18]. Hardy’s line of work challenged the multi-dimensional versus unitary structure of MT by showing that commonly used MT attributes overlapped substantially, limiting discriminant validity and supporting a direct unidimensional model across sport, academic, and business groups [19]. Gucciardi acknowledged that, despite hundreds of studies, concerns remain about both conceptualization and measurement, and that substantive and methodological work is still needed to refine the construct and build theory [10,20,21]. The MTI was selected for the present study because its brief, state-sensitive design targets a narrower, performance-context-specific resource compared with earlier multi-dimensional inventories (e.g., MTQ48 and SMTQ), providing a partial response to discriminant validity concerns. Nevertheless, unresolved conceptual overlap with self-efficacy and resilience remains a theoretical limitation that should be considered when interpreting MTI-based findings. Despite these ongoing theoretical debates, the state-sensitive MTI provides a tractable operationalization for studying MT fluctuation in demanding sport contexts.

Rowing exemplifies such a discipline, requiring athletes to maintain technical precision and psychological control under prolonged physical strain. It has been recognized as a sport that demands sustained physical exertion while requiring the ability to manage discomfort over extended periods of time [22,23]. Rowing success is predicated on a complex interplay of physiological attributes, biomechanical efficiency, and psychological fortitude [24]. Rowing, with its unique combination of high-intensity bursts and prolonged periods of sustained power output, presents a particularly salient context for the influence of psychological attributes, such as MT [25,26]. The ability to persevere through intense physical demands, maintain pacing strategies, and cope with competitive stressors may plausibly be related to MT, although the size and direction of this relationship require longitudinal examination [4,27,28,29,30,31].

Despite the intuitive appeal of MT as a key factor in rowing performance, the existing body of empirical research specifically investigating this relationship remains relatively sparse. A significant proportion of the limited research that has been conducted in this area has relied on cross-sectional study designs, providing a valuable but limited snapshot of the association between MT and performance at a single point in time [2,26,27,32]. While these studies offer initial insights, they are inherently limited in their ability to capture the dynamic and longitudinal nature of the relationship between MT and performance as it unfolds across a competitive season. A more nuanced understanding of this relationship requires longitudinal, repeated-measures designs capable of tracing how fluctuations in MT correspond with variations in performance over time [33].

Collegiate rowing, particularly within the Division II athletic landscape, provides a compelling and ecologically valid context for investigating the interplay between psychological factors and athletic achievement. Off-water rowing performance is typically assessed by the 2000 m (2K) time trial. This trial is used as a fundamental and standardized assessment of a rower’s physiological capacity, pacing efficacy, and psychological regulation under effort [30,34]. This maximal effort test, typically conducted on an indoor rowing ergometer under controlled conditions, is a critical benchmark used by coaches for athlete evaluation, boat selection, and the monitoring of individual progress throughout the training year [30,34,35]. Consequently, identifying the psychological factors that significantly contribute to success in this pivotal performance indicator holds substantial practical relevance for coaches seeking to optimize athlete development and enhance overall team performance [36].

A female-specific longitudinal study is scientifically justified because female athletes face sex-linked physiological fluctuations and psychosocial contexts that can alter both performance-related states and the way MT relates to outcomes [37,38]. In comparison to their male counterparts, female athletes report more anxiety, depression, distress, and disordered eating, indicating a distinct mental-health context for performance research [39]. In addition, the effects of cyclical hormonal variation across the menstrual cycle on performance modulation and psychological state variables are mixed rather than uniform [37,40,41,42,43,44,45,46]. A female-specific longitudinal investigation is, therefore, warranted as an independent scientific contribution, not merely as a representational corrective.

Given the gaps in longitudinal evidence linking MT to rowing performance [33], limited female representation [47,48], and the recognized significance of the 2K time trial as a key performance metric in collegiate rowing [30,49,50], the present longitudinal observational follow-up study evaluated: (a) the between-athlete question (‘are tougher athletes faster?’), and (b) the within-athlete question (‘is an athlete faster on days when they are tougher than usual?’). These are conceptually distinct estimands that can yield different and independently important answers, but prior cross-sectional studies often conflate these two questions. The longitudinal within–between decomposition employed here explicitly separates them, making the within-athlete estimand the primary target of inference. We hypothesize that timepoints with higher within-athlete MTI results would be associated with faster (lower) 2K times, while acknowledging that stable between-athlete differences are likely to account for substantial performance variability.

## 2. Materials and Methods

### 2.1. Participants

Twelve female collegiate rowers (age: 20.8 ± 2.1 years; training experience: 2.8 ± 3.0 years) from a Division II university program voluntarily participated in this longitudinal observational study. The sample comprised the entirety of the university’s roster, constituting a convenience cohort, a pragmatic approach common in sport science research involving specialized athletic populations [51,52,53]. Athletes were healthy and accustomed to rigorous training, shared a comparable background in competitive rowing, and standardized 2K time-trial procedures. Ethical approval was secured via the Institutional Review Board (#1968553-3), with written informed consent obtained from all athletes. Consent documentation clarified participation risks, benefits, and voluntary withdrawal rights, ensuring compliance with the Declaration of Helsinki principles. All 12 athletes completed every assessment across the season, resulting in a balanced dataset with no missing observations.

### 2.2. Study Design and Procedures

In this longitudinal observational follow-up study, data on each rower’s MT and 2K rowing performance were collected across four distinct time points during a single competitive season. Data collection occurred on four calendar dates (2 February, 15 February, 4 March, and 5 April 2023), corresponding to intervals of 13, 18, and 32 days between sessions, as part of their official team testing, embedding this study seamlessly into existing training cycles and competition. This alignment aimed to capture fluctuations across distinct seasonal phases (e.g., preseason conditioning, midseason, and championship tapering), while avoiding disruptive protocol deviations.

At each session, the participants first completed the MTI in a controlled laboratory environment, minimizing external distractions. Standardized verbal instructions preceded the questionnaire, emphasizing candid responses to how athletes perceived themselves at the assessment point in the context of their current training and performance demands. Immediately afterward, the athletes performed a 2K time trial on a calibrated Concept2 Model D ergometer (Concept2, Inc., Morrisville, VT, USA), a protocol repeated identically across all timepoints [35,50]. Environmental conditions (temperature: 21–23 °C; humidity: 50–60%) and pre-test instructions were rigorously standardized. The athletes were instructed to perform a maximal effort to replicate race intensity, with completion time recorded automatically by the ergometer monitor to the nearest 0.1 s. 2K time served as the primary performance outcome and was also used for team monitoring (e.g., selection decisions). To reduce response bias, the MTI questionnaires were completed privately under researcher supervision with coaching staff absent; individual MTI responses were not shared with coaches and were not linked to performance feedback.

### 2.3. Mental Toughness Index (MTI)

Mental toughness was quantified using the 8-item MTI [11], a validated self-report measure intended to capture a generalized psychological resource for persisting and performing under pressure and adversity (see Supplementary Material). Items (e.g., “I overcome setbacks without undue frustration”) are rated on a 7-point Likert scale (1 = False, 100% of the time; 7 = True, 100% of the time) and summed to yield a total score ranging from 8 to 56, with higher scores indicating greater MT. The MTI demonstrates robust psychometric properties, including internal consistency (α = 0.80–0.90) and discriminant validity across athletic cohorts [54,55,56,57]. Its brevity made it practical for repeated assessment in time-constrained athletes and suitable for examining whether MTI scores varied within athletes across the season. In the present cohort, the MTI total scores demonstrated moderate test–retest stability across the four timepoints (ICC [1,1] = 0.66). This indicates that while MT possesses a stable trait component, an ICC of 0.66 is consistent with the state-like framing of the MTI: moderate stability confirms that person–mean decomposition is meaningful (athletes differ systematically in average MT), while ~34% within-person variance ensures the within-athlete estimand is non-trivial and not purely noise. A near-zero ICC would render a within-athlete analysis uninformative; a near-unity ICC would leave no within-athlete variation to model. The intermediate value is analytically appropriate for the within–between design. However, ICC [1,1] = 0.66 also conflates true state change with measurement error; some portion of the observed 34% within-athlete variance reflects instrument unreliability rather than genuine psychological fluctuation. This measurement-error component would attenuate the within-athlete estimate (β within) toward zero and should be treated explicitly as an additional limitation of the precision of the within-athlete association. Because item-level MTI responses were not retained, internal consistency (α/ω) could not be computed for this sample and is, therefore, reported from prior validation studies (typically α = 0.80–0.90).

### 2.4. 2K Rowing Performance

Performance was operationalized as time-to-completion (seconds) on the Concept2 Model D ergometer, a widely used standardized field test in collegiate rowing [23]. The 2K trial is routinely used for athlete monitoring and selection and has been reported to correlate with on-water performance (*r* = 0.72–0.89) [30,49,58,59]. Protocol adherence included a 10 min standardized warm-up, consistent drag factor settings, seat adjustments for anthropometric consistency, and immediate post-test cooldown. This meticulous replication across testing waves minimized instrumentation bias, ensuring data comparability. Based on established sport-science standards for elite and collegiate rowers, the typical error of measurement between repeated 2K ergometer trials is approximately 0.5% (~2.5 s for a 500 s trial), with the threshold for a baseline Smallest Worthwhile Change (SWC) set at 0.3% (~1.5 s) [60,61].

## 3. Statistical Analysis

The primary analytical objective was to estimate the longitudinal relationship between MT, operationalized via the MTI score, and 2K rowing performance, measured in seconds, across a competitive season. Consistent with contemporary recommendations to prioritize precision-based estimation over null-hypothesis significance testing in sport and exercise science [51,62,63,64,65], all primary analyses report point estimates with 95% confidence intervals as the principal inferential statistic, with *p*-values reported as secondary descriptive indices. No a priori power analysis was conducted because the cohort was fixed by the team roster; we emphasize the estimation (CIs) and treat findings as preliminary. The dataset comprised 48 observations (four timepoints) nested within 12 athletes, creating a repeated-measures structure with non-independence of observations within athletes. Accordingly, analyses used a linear mixed-effects modeling (LMEM) framework with a random intercept for the athlete to account for within-athlete clustering and stable baseline performance differences [66,67,68,69]. All analyses were conducted in R (v4.3.1), using lme4 for model estimation and clubSandwich for small-sample cluster-robust inference. The models were estimated using the restricted maximum likelihood (REML) [70].

### 3.1. Model Specification

To separate stable between-athlete differences in MT from within-athlete fluctuations over the season, the MTI was decomposed into:

- MTI_between_: Each athlete’s season-average MTI, representing stable between-athlete differences in average MTI across the study period (between-athlete/trait component).
- MTI_within_: Each observation’s deviation from that athlete’s season-average MTI, representing timepoint-specific within-athlete deviations in the MTI (within-athlete/state component).

The timepoint was modeled as a categorical fixed effect (Reference = Baseline/Timepoint 1) to allow non-linear seasonal patterns and to adjust for season phase effects (e.g., early-, mid-, late-season training periods). Categorical coding was preferred over continuous-time modeling because the four sessions were separated by non-uniform intervals (13, 18, and 32 days) and corresponded to qualitatively distinct seasonal phases as selected by the coaching and the related NCAA scheduling. Researchers had no control over session timing, and imposing a linear or polynomial time trend would presuppose an unverified functional form on these non-equidistant occasions. Treating the four sessions as categorical phases is justified when the observation gaps are uneven and the occasions are substantively distinct, because mixed-model specification should be driven by the design and by what the data can support rather than by an imposed functional form [71,72]. Specifically, the inability to estimate random slopes in the present design reflects both the small number of athlete clusters (N = 12) and the limited number of repeated observations per participant (four timepoints); with only four observations per athlete, a random-slope model would be severely underdetermined. A random-intercept specification is defensible only as a parsimonious choice when richer random-effects structures are not supported by the design or data, since random-effects complexity trades off Type I error control against power [72]. The primary model specification was:

2Kij = β_0_ + βT2 I(T2) + βT3 I(T3) + βT4 I(T4) + βB (MTI_between_,j) + βW (MTI_within_,ij) + u0j + εij

where u0j is the athlete-specific random intercept, and εij is the within-athlete residual. This specification targets the within-athlete estimand (βW): the expected difference in 2K time associated with an athlete being higher or lower than their season average at a given timepoint, adjusted for the season phase and stable between-athlete differences.

### 3.2. Small-Sample Robust Inference

Because the number of clusters was small (*N* = 12), conventional LMEM standard errors can yield unreliable uncertainty estimates. Therefore, inference for fixed effects relied on CR2 cluster-robust standard errors clustered by athlete with Satterthwaite degrees of freedom, producing robust 95% confidence intervals and *p*-values [70]. The results are reported as fixed-effect estimates (*β*), robust 95% CIs, and *p*-values, with interpretation emphasizing effect magnitude and precision (interval estimates) consistent with pilot/special-population aims.

### 3.3. Sensitivity Analysis

To evaluate the robustness of the primary within-athlete MTI estimate and reduce dependence on any single athlete, we applied the following:

- Leave-One-Out (LOO) Analysis: The primary model was refit 12 times, omitting one athlete per iteration; the range of βW estimates was summarized to assess the direction and stability of the association.
- Unadjusted Model: An unadjusted random-intercept model was fit to provide a comparator consistent with cross-sectional or minimally adjusted approaches 2K~MTI + (1|Athlete).

### 3.4. Diagnosis and Variance Decomposition

Assumptions were evaluated primarily via graphical diagnostics (residuals vs fitted values, Q–Q plots) and influence checks; given the small sample, diagnostics were interpreted descriptively rather than relying on formal normality tests. Fixed-effect collinearity was assessed using Variance Inflation Factors (VIFs) from the fixed-effects design matrix; all VIFs were low (≤1.58), supporting the inclusion of timepoint and the within–between MTI terms. To quantify the extent to which performance differences reflected stable between-athlete differences versus within-athlete fluctuation, we computed the Intraclass Correlation Coefficient (ICC) and Nakagawa’s Marginal and Conditional *R*^2^ [73].

## 4. Results

### 4.1. Descriptive Statistics and Model Assumptions

Descriptive statistics for MTI scores and 2K performance across the four timepoints (*N* = 48 observations; *n* = 12 at each timepoint for both MTI and 2K; and no missingness) are presented in Table 1. The cohort demonstrated a seasonal improvement in 2K performance, with mean 2K time improving by approximately 8.9 s from baseline (499.6 s) to the final assessment (490.7 s). Concurrently, the mean MTI scores increased from 42.5 at baseline to 46.2 at the final timepoint.

**Figure 1:**
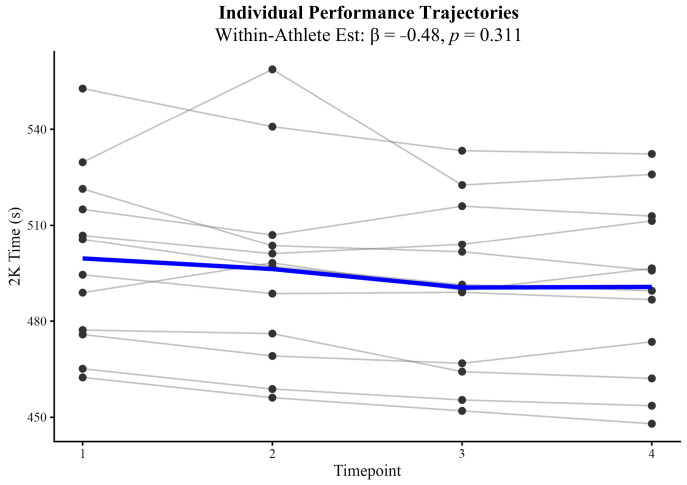
Individual 2K rowing performance trajectories across the competitive season. Note: The grey lines represent individual athletes (*N* = 12). The blue line represents the group means at each timepoint. The subtitle displays the estimated within-athlete association between mental toughness and 2K time from the primary mixed model.

**Figure 2:**
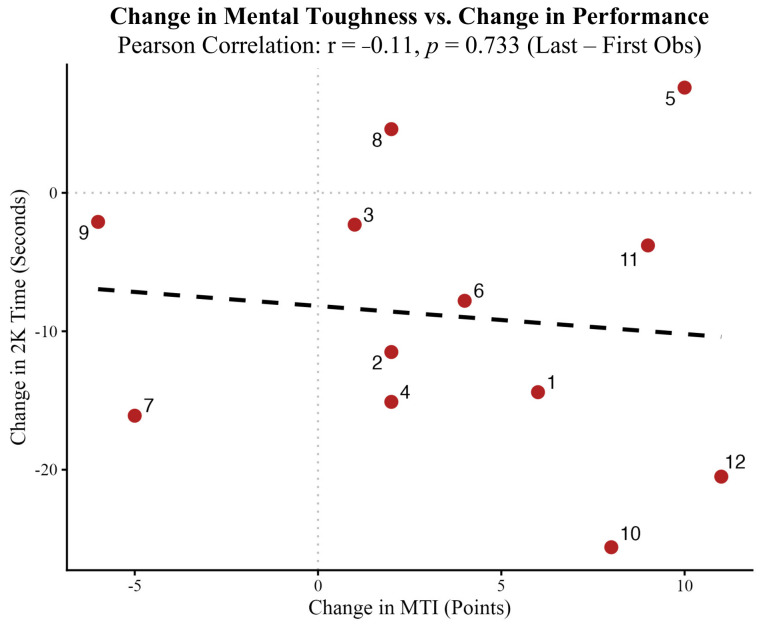
The association between change in the mental toughness index (MTI) and change in 2K rowing performance. Note: Each point represents one athlete, labeled by ID. Change scores are calculated as the last observation minus the first observation. The dashed line represents the linear regression trend. The points in the bottom-right quadrant indicate athletes who increased their mental toughness and improved their performance (faster time).

Athlete-level heterogeneity was evident across timepoints (Figure 1) and in baseline-to-final change scores (Table 2; Figure 2; see Supplementary Figures S1 and S2): 10/12 athletes improved (negative Δ2K), while 2/12 worsened. For example, while Athlete 10 improved their 2K time by 25.6 s, Athlete 9 showed a minimal change (−2.1 s). Similarly, MTI changes ranged from +11 points (Athlete 12) to −6 points (Athlete 9). Fixed-effect collinearity was low (all VIFs ≤ 1.58), supporting simultaneous inclusion of the timepoint and the within–between MTI terms. Visual inspection of residual and *Q*–*Q* plots indicated no severe deviations from normality (Supplementary Figure S3), and residual-versus-fitted patterns did not suggest gross heteroscedasticity (see diagnostics panel in the Supplementary Materials).

### 4.2. Linear Mixed-Effects Model Analyses (CR2 Robust Inference)

#### Primary Model: Time-Adjusted Within–Between MTI (Random Intercept for Athlete)

The primary linear mixed-effects model, adjusted for the seasonal phase and stable between-athlete differences, revealed clear descriptive improvements in performance over the season (Table 3). Specifically, performance at Timepoint 3 was 7.3 s faster than baseline (*β* = −7.29 s, *p* = 0.023), indicating a timepoint-related performance difference relative to baseline. The primary estimand, the within-athlete association between MTI and performance, was estimated at *β* = −0.48 s per the MTI point (Robust 95% CI [−1.56, 0.59], *p* = 0.311). This indicates that each 1-point higher-than-usual MTI score was associated with an approximately 0.48 s faster 2K time, although the confidence interval was wide and included slower times. Per contemporary recommendations to interpret confidence intervals as compatibility intervals rather than as binary significance tests [62,74], this confidence interval is most accurately characterized as encompassing a range of compatible effects from approximately 1.6 s faster to 0.6 s slower per MTI point. The compatibility interval spans both negative and positive values, indicating substantial uncertainty in both the direction and magnitude of the association; the result is, therefore, most appropriately described as inconclusive rather than null [75,76,77,78].

### 4.3. Variance Decomposition and Sensitivity

Variance decomposition indicated that 2K performance was dominated by stable between-athlete differences, which accounted for 94.6% of the variance (ICC = 0.946). The full model explained 94.8% of the total variance (Conditional *R*^2^ = 0.948). In contrast, the fixed effects (season + MT) explained approximately 3.3% of the variance (marginal *R*^2^ = 0.033). Sensitivity analyses confirmed that no single athlete drove the within-athlete MTI estimate, while reaffirming that the estimate remained consistently imprecise and inconclusive across all refits:

- Leave-One-Out (LOO): Iteratively removing one athlete at a time resulted in consistent negative coefficients for the within-athlete MTI effect (range: *β*_within_ = −0.79 to −0.25).
- Unadjusted Model: When the effect of time was removed from the model (sensitivity model), the association between the MTI and performance appeared stronger (*β* = −0.96 s).

However, attenuation of the MTI estimate after adjusting for the timepoint suggests that the unadjusted association may partly reflect season progression or other time-linked factors, supporting the time-adjusted primary model. Sensitivity analyses showed that the within-athlete MTI estimate was consistently negative across all 12 leave-one-out refits, suggesting that no single athlete fully drove the negative point estimate; this leave-one-out analysis is a robustness/influence check only and does not constitute evidence against a true null effect. The unadjusted sensitivity model (time 2k~MTI, without timepoint control) yielded *β* = −0.96 s, 95% CR2 CI [−1.90, −0.03], *df* Satt = 6.22, *p* = 0.045. Notably, this unadjusted CI excludes zero, contrasting with the primary adjusted model CI [−1.56, 0.59], which spans zero, directly supporting the importance of controlling for the seasonal timepoint [65,68,76,77,78].

## 5. Discussion

This pilot longitudinal study evaluated whether MT, operationalized via the MTI, covaried with 2K ergometer performance across a competitive season in a special population of collegiate female rowers. Using a parsimonious mixed-effects model with within–between MTI decomposition and small-sample CR2-robust inference, the results indicate that (a) 2K performance improved mid-season relative to baseline; (b) stable between-athlete differences dominated over variability (ICC = 0.95); and (c) within-athlete MTI deviations showed a small, negative association with performance (*β*_within_ = −0.48 s per MTI point) that was imprecisely estimated after adjusting for timepoint. Accordingly, and following contemporary recommendations for precision-based inference in sport and exercise science [51,53,65], we interpret these results as statistically inconclusive and, per contemporary estimation-focused frameworks, as hypothesis-generating rather than confirmatory, within this pilot cohort. The data neither establish MT as a driver of 2K performance nor as practically negligible, and they should not be classified as a null result in the technical sense [62,74].

### 5.1. Interpretation of the Findings

Cross-sectional associations between MT and performance may partly reflect stable athlete differences and season progression rather than within-athlete covariation [4,7,33]. In the present longitudinal within–between decomposition, the primary estimand was the within-athlete association, whether an athlete performs better than usual when they report being tougher than usual [79]. The CR2-robust estimate suggested a small association (*β*_within_ = −0.48 s per MTI point; 95% robust CI [−1.56, 0.59]; *p* = 0.311), indicating substantial uncertainty in magnitude in this pilot cohort [51]. From a practical standpoint, a 5-point higher-than-usual MTI score was associated with an estimated ~2.4 s faster (5 × 0.48 s; given *β*_within_ is negative), though the compatible range remains wide (i.e., −7.8 to +3.0 s) given the CI. In 2K rowing, where close finishes can be decided by small margins [23,34,49], even modest effects could be meaningful for some athletes, but larger samples with richer covariate measurement are needed to confirm the size and consistency of this within-athlete relationship.

### 5.2. Comparison to the Existing Literature

The direction of association observed here, higher MT corresponding to faster performance on average, is broadly consistent with the prior literature linking MT to endurance-related performance and persistence under discomfort [4,7,80,81,82]. However, the present findings also underscore that the magnitude of association can depend strongly on study design and analytic approach. In our pilot cohort, the association attenuated after adjusting for the timepoint (season progression) and separating within-athlete deviations from stable between-athlete differences, indicating that some cross-sectional or minimally adjusted relationships may reflect stable athlete characteristics and concurrent season effects rather than within-athlete covariation [33]. Evidence across studies is mixed, which may reflect differences in how MT is operationalized (e.g., MTI vs. other instruments), performance contexts, and whether analyses distinguish within-athlete change from between-athlete differences [11,26]. The within–between decomposition used here specifically targets whether an athlete performs better than usual when they report being tougher than usual, which is conceptually distinct from comparing athletes who differ in average mental toughness and may yield smaller effects [79]. If MT is related to 2K performance within athletes, plausible pathways may include effort regulation under discomfort, attentional control, and adherence to pacing strategy during maximal effort, operating alongside (and interacting with) physiological capacity, technical proficiency, and training load [23,31,36]. Reverse temporal ambiguity should also be acknowledged: athletes performing well (recovered, healthy, and at peak fitness) may self-report higher MT as a consequence of feeling capable, so observed within-athlete co-movement could partly run performance → MT rather than MT → performance; future designs should consider lagged analyses to address directionality. Additionally, consistent with network-physiology perspectives on dynamic interactions among performance-relevant systems, the present findings highlight that MTI–performance associations may not be uniform across athletes and should be interpreted alongside athlete-specific trajectories rather than only through a single group-level estimate [50].

### 5.3. Methodological Strengths

A key strength of this study is the complete repeated-measures design (48/48 observations), which allowed evaluation of change within athletes across four standardized assessments embedded in routine team testing [33]. Analytically, the random-intercept mixed-effects framework appropriately accounts for the non-independence of repeated observations within athletes and quantifies stable between-athlete differences in baseline performance [83]. Importantly, we used a within–between decomposition of the MTI to distinguish whether athletes perform better when they report being tougher than usual (within-athlete deviations) from stable between-athlete differences in average MTI [79]. Given the small number of athletes (clusters), we emphasized estimation and uncertainty using CR2 cluster-robust standard errors and robust confidence intervals, complemented by leave-one-athlete-out sensitivity analyses [51,66]. Finally, we paired model-based estimates with case-series tables and individual trajectory figures to improve transparency and practical interpretability for coaches working with small teams. These combined elements (robust estimation + diagnostics + individual trajectories) support a quantitative case-series interpretation appropriate for small, specialized athletic cohorts [4,27].

### 5.4. Limitations

While these findings offer preliminary insight into a potential exploratory role of MT in rowing, several limitations warrant consideration. First, the sample was small (*N* = 12) and drawn from a single Division II women’s program, limiting precision and generalizability to other levels or populations. We addressed small-sample uncertainty by using CR2 cluster-robust inference, emphasizing confidence intervals and effect estimation rather than relying on null-hypothesis significance testing [51,66]. Second, the homogeneity of the sample with respect to sex, competitive level, and institutional affiliation may limit the generalizability of the findings to other populations of rowers, including male athletes, individuals competing at different levels (e.g., Division I, club rowing, or elite international competition), and rowers from diverse cultural backgrounds and training environments [25,27,31,81]. Third, this study was observational. Although models adjusted for the timepoint (season progression), time-varying factors (e.g., training load/recovery, illness/injury, physiological adaptation, sleep/nutrition, athlete-coach dynamics, motivational climate, and burnout) may have influenced both the MTI and performance, and residual confounding cannot be excluded; therefore, effect estimates should be interpreted as associative [23,27,84,85]. Fourth, MT was assessed via self-reported MTI. While the MTI is a widely used and psychometrically sound instrument, the use of repeated self-report questionnaires inherently introduces the potential for various response biases, such as social desirability bias, where participants may consciously or unconsciously tend to present themselves in a more socially desirable or mentally tough manner. Although the MTI totals showed moderate stability across the season (ICC = 0.66), item-level responses were not retained, preventing internal consistency estimation (*α*/*ω*) in this cohort; repeated self-report also remains susceptible to response biases (e.g., socially desirable responding) [11,86,87]. Critically, because item-level MTI responses were unavailable, the degree to which within-athlete variance reflects genuine psychological fluctuation versus measurement error cannot be quantified. Attenuation due to measurement error is expected to bias the within-athlete estimate toward the null. Consequently, both the magnitude and the precision of the estimated within-athlete association remain uncertain, and the inconclusive finding cannot be interpreted as evidence of absence of an effect [88,89,90]. Finally, performance was assessed using ergometer 2K time trials; while standardized and practically relevant, this outcome does not capture on-water technical efficiency, tactical demands, or boat dynamics that also contribute to competitive success [23,26,30,33,34,49,91]. A further limitation pertains to the absence of the qualitative depth that a prospective multiple longitudinal case study approach would provide, as such designs are uniquely positioned to answer “how” and “why” questions through contextual qualitative interpretation [92,93]. This study was approved as a quantitative longitudinal observational design to address a research question regarding the association between within-athlete MTI fluctuations and 2K performance after accounting for season progression. In essence, we sought to investigate the “how much/under what conditions” question for which mixed-effects modeling was deemed the most methodologically suitable framework to address the research question [79,83]. Despite this limitation, this manuscript provides substantial individual-level transparency, establishing what is termed “replication logic across cases” within a quantitative framework [93]. Of particular importance for a study of female athletes, menstrual cycle phase and hormonal contraceptive status were not assessed, despite their established capacity to co-vary with both psychological state and maximal aerobic performance across a multi-week window. While these time-varying physiological metrics are rarely captured during routine collegiate monitoring, their potential contribution to within-athlete performance variance warrants prospective measurement in future designs [6,40,42,44,94,95]. Group-level performance effects of the menstrual cycle on objective aerobic metrics are often small or inconsistent, yet individual psychological states and performance capacities can vary dynamically across phases [43,45,46]. Because oral contraceptive influences are similarly heterogeneous rather than uniform [42,44], unmeasured cycle dynamics across our 2-month testing window (February–April 2023) plausibly introduced residual within-athlete variance that our fixed-effects structure could not isolate. Furthermore, the training load warrants emphasis beyond mere mention as a missing covariate: given that fixed effects explain only ~3.3% of variance (R^2^_m_ = 0.033) while stable between-athlete differences account for 94.6% (ICC = 0.946), cumulative training adaptation likely contributed to the performance improvement at Timepoints 3–4, and its omission complicates interpretation of the within-athlete MTI estimate.

### 5.5. Future Research Directions

The single most critical design priority for future studies is the prospective monitoring of the training load and physiological recovery alongside repeated MT assessment. Session-RPE, HRV-based recovery indices, sleep duration, and GPS load metrics would allow researchers to determine whether the within-athlete MTI association persists after controlling for fluctuations in physical readiness; this confounder is most likely responsible for residual within-athlete variance in the present design.

Future research should replicate these preliminary estimates in larger, multi-site samples of rowers (including men and athletes across competitive levels) to improve precision and external validity [8,27,33]. A priority is to pair repeated MT assessment with key performance determinants that were not available here, training load and recovery indices (as specified above), physiological markers (e.g., aerobic capacity/lactate metrics), and technical efficiency measures, to better address time-varying confounding and clarify when MT adds incremental value beyond season progression [12,23]. Longer follow-up designs spanning multiple training phases would also help distinguish within-athlete MTI variation from more stable between-athlete differences [33]. Methodologically, intervention studies (e.g., randomized or pragmatic trials of mental skills training) are needed to test whether enhancing MT produces measurable improvements in 2K and/or on-water performance [2,11,92,96]. Future work would also benefit from multi-method MT measurement (e.g., retaining item-level responses to estimate internal consistency in-sample, incorporating behavioral tasks or coach ratings, and qualitative interviews) to triangulate construct validity and reduce sole reliance on repeated self-report [3,4,11,12,32,81,97].

In addition, future longitudinal work in small specialized athletic cohorts would benefit substantially from a prospective mixed-methods or multiple longitudinal case study design [92,93] that integrates: (a) pre-registered analytical and qualitative protocols on the Open Science Framework prior to data collection [65,98] to protect against HARKing and analytical pivoting [99]; (b) qualitative interview schedules administered alongside MTI completion at each timepoint to capture athlete narratives, perceived stressors, and meaning-making processes; (c) systematic coaching observations using validated rubrics to capture coaching context and inter-rater perspectives; (d) prospective training load and recovery monitoring (session-RPE, sleep duration and quality, and heart rate variability) to address time-varying confounding; and (e) injury and illness diaries to capture intercurrent events that may simultaneously influence MT and performance. Such a design would provide the individual-level explanatory depth that purely quantitative models of this size cannot achieve, while preserving the analytical transparency and replicability that contemporary sport science scholarship has identified as essential [53,100,101]. Critically, the prospective specification of both quantitative and qualitative elements may permit rigorous integration of within-athlete trajectory modeling with contextual case-level interpretation.

### 5.6. Practical Implications

**Figure 3:**
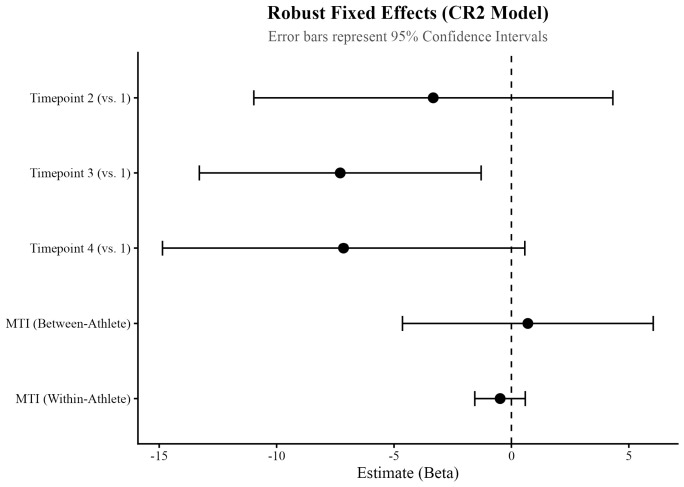
Robust fixed-Effect estimates and 95% confidence intervals from the primary linear mixed-effects model. Note: The error bars represent 95% confidence intervals computed using cluster-robust (CR2) variance estimation. Estimates crossing the vertical dashed line (0) indicate that the compatibility boundaries encompass a zero effect.

Important interpretive context: this was an observational study in which no manipulation of MT was attempted, and no causal inference about the effect of mental skills training on rowing performance is warranted. All practical discussion that follows is therefore conditional, hypothesis-generating, and must be interpreted accordingly. These pilot data suggest that the within-athlete point estimate for MT are associated with faster rowing times, though the robust 95% CI spans both practically meaningful effects—the lower compatibility bound of −1.56 s/point implies a 5-point MTI shift of ~7.8 s, exceeding the established SWC of ~1.5 s [60,61]—and negligible or opposing effects (the upper bound of +0.59 s/point implies performance deterioration). Because the compatibility boundaries straddle this practical threshold, the primary within-athlete estimand must be interpreted as statistically inconclusive. Any practical role for mental skills development in rowing as a performance complement remains to be established in adequately powered, covariate-informed longitudinal studies. In an unadjusted model, a 5-point MTI difference corresponded to an estimated ~5 s in 2K time; however, this association attenuated after adjusting for the timepoint (season progression) and separating within- from between-athlete components. In the primary CR2-robust within–between model, the within-athlete estimate was small and imprecise, indicating that the magnitude of any marginal benefit remains uncertain in this cohort. Given that stable between-athlete differences dominated performance variability, individualized monitoring of both performance trajectories and psychological state across the season (Table 2; Figure 1, Figure 2 and Figure 3) may be more actionable for coaches than relying solely on group-level inference in small teams [27]. Although this study does not establish that increasing MT causes faster performance, the observed direction of association is consistent with the possibility that MT may function as a performance-relevant psychological resource under discomfort, particularly in contexts where competitive margins are small. In race settings where external input is limited, and athletes must sustain effort and pacing under fatigue, mental skills that support attentional control, coping, and effort regulation may be useful adjuncts to training [23,36]. More generally, coaches and practitioners may consider integrating mental skills development into existing training programs (e.g., goal setting, imagery, self-talk routines, mindfulness/attention strategies, and coping plans for setbacks), implemented alongside structured physical conditioning and technical work. The most practical use in small squads may be to track individual responses over time and tailor support to athletes who show meaningful co-movement between psychological state and performance [3,4,27,33,36,96,102].

## 6. Conclusions

In conclusion, in this small special-population cohort of Division II female rowers assessed repeatedly across a season, between-athlete differences accounted for the vast majority of 2K performance variance, while the MTI showed a small, imprecise, and inconclusive within-athlete association and must be treated as exploratory and hypothesis-generating, pending replication. The magnitude of any complementary role that MT or MT-related mental performance factors into rowing performance cannot be reliably estimated from this pilot dataset alone, and the inconclusive results should not be interpreted as either confirming or dismissing that role. Consequently, researchers and practitioners should move beyond cross-sectional comparisons and prioritize individualized, covariate-informed longitudinal monitoring of the MTI and other psychological performance indicators alongside training and performance indicators to clarify when and for whom MT meaningfully relates to rowing performance.

## Figures and Tables

**Table 1:** Descriptive statistics for the mental toughness index (MTI) and 2K rowing performance across the four timepoints.

Timepoint	MTI Score	2K Time (Seconds)
1	42.5 (6.1)	499.6 (27.5)
2	42.5 (6.1)	496.3 (30.6)
3	46.2 (4.0)	490.5 (26.6)
4	46.2 (6.7)	490.7 (27.4)

Note: *N* = 12 for all timepoints. Values are presented as mean (standard deviation). SD—standard deviation; 2K—2000 m; MTI—mental toughness index.

**Table 2:** Individual athlete performance, mental toughness trajectories, and change scores.

Athlete	2K (First)	2K (Last)	MTI (First)	MTI (Last)	Δ 2K (s)	Δ MTI
1	462.4	448.0	44.0	50.0	−14.4	6.0
2	465.1	453.6	48.0	50.0	−11.5	2.0
3	475.8	473.5	30.0	31.0	−2.3	1.0
4	477.2	462.1	45.0	47.0	−15.1	2.0
5	488.9	496.5	32.0	42.0	7.6	10.0
6	494.5	486.7	47.0	51.0	−7.8	4.0
7	505.6	489.5	45.0	40.0	−16.1	−5.0
8	506.8	511.4	41.0	43.0	4.6	2.0
9	515.0	512.9	50.0	44.0	−2.1	−6.0
10	521.4	495.8	44.0	52.0	−25.6	8.0
11	529.7	525.9	39.0	48.0	−3.8	9.0
12	552.8	532.3	45.0	56.0	−20.5	11.0

Note: The first and last indicate the first and last valid observation for each athlete. Δ indicates the difference (last–first). A negative Δ 2K value represents a performance improvement (faster time). 2K—2000 m; and MTI—mental toughness index.

**Table 3:** The results of the linear mixed-effects model predicting 2K rowing performance (seconds) with robust standard errors.

Predictor	Estimate	SE	*df* (Satt)	95% CI	*p*
Intercept (MTI = 0) ^a^	498.71	7.81	9.84	[481.27, 516.14]	<0.001
Timepoint 2 (vs. 1)	−3.33	3.47	11.00	[−10.97, 4.32]	0.359
Timepoint 3 (vs. 1)	−7.29	2.61	8.19	[−13.29, −1.29]	0.023
Timepoint 4 (vs. 1)	−7.15	3.23	6.68	[−14.86, 0.57]	0.064
MTI (Between-Athlete)	0.70	1.44	2.38	[−4.64, 6.04]	0.667
MTI (Within-Athlete)	−0.48	0.43	5.70	[−1.56, 0.59]	0.311

Note: SE = standard error (cluster-robust CR2). CI = confidence interval. The model included a random intercept for the Athlete. ‘Within-athlete’ refers to deviations from an athlete’s own mean MTI; ‘between-athlete’ refers to the athlete’s average MTI. ^a^ MTI_between_ centered at the grand mean (M = 44.35); and intercept = predicted 2K time for an athlete at the average between-athlete MTI (verified: *β* = 498.71 s, SE = 7.81, 95% CI [481.27, 516.14]).

## Data Availability

The datasets generated and/or analyzed during the current study are publicly available on the Open Science Framework (OSF) at https://doi.org/10.17605/OSF.IO/ZF3RQ. All data have been de-identified to protect participant confidentiality and comply with institutional ethical standards.

## Supplementary Materials

The following supporting information can be downloaded at: https://www.mdpi.com/article/10.3390/sports14070282/s1, Figure S1: 2K Distribution; Figure S2: MTI Distribution; Figure S3: Q-Q Plot.

## Abbreviations

The following abbreviations are used in this manuscript:

MT	Mental toughness
MTI	Mental toughness index
NCAA	National Collegiate Athletics Association
2K	2000 m
ICC	Intraclass Correlation Coefficient
REML	Restricted maximum likelihood
LOO	Leave-one-out
VIFs	Variance Inflation Factors
