# Supplementary figures and images for: Mental Toughness and 2K Rowing Performance in Division II Female Collegiate Athletes: A Longitudinal Analysis Using Mixed-Effects Modeling
Source: Sports (Basel). 2026 Jul 6;14(7):282. doi: 10.3390/sports14070282 (PMC13417108; doi:10.3390/sports14070282)

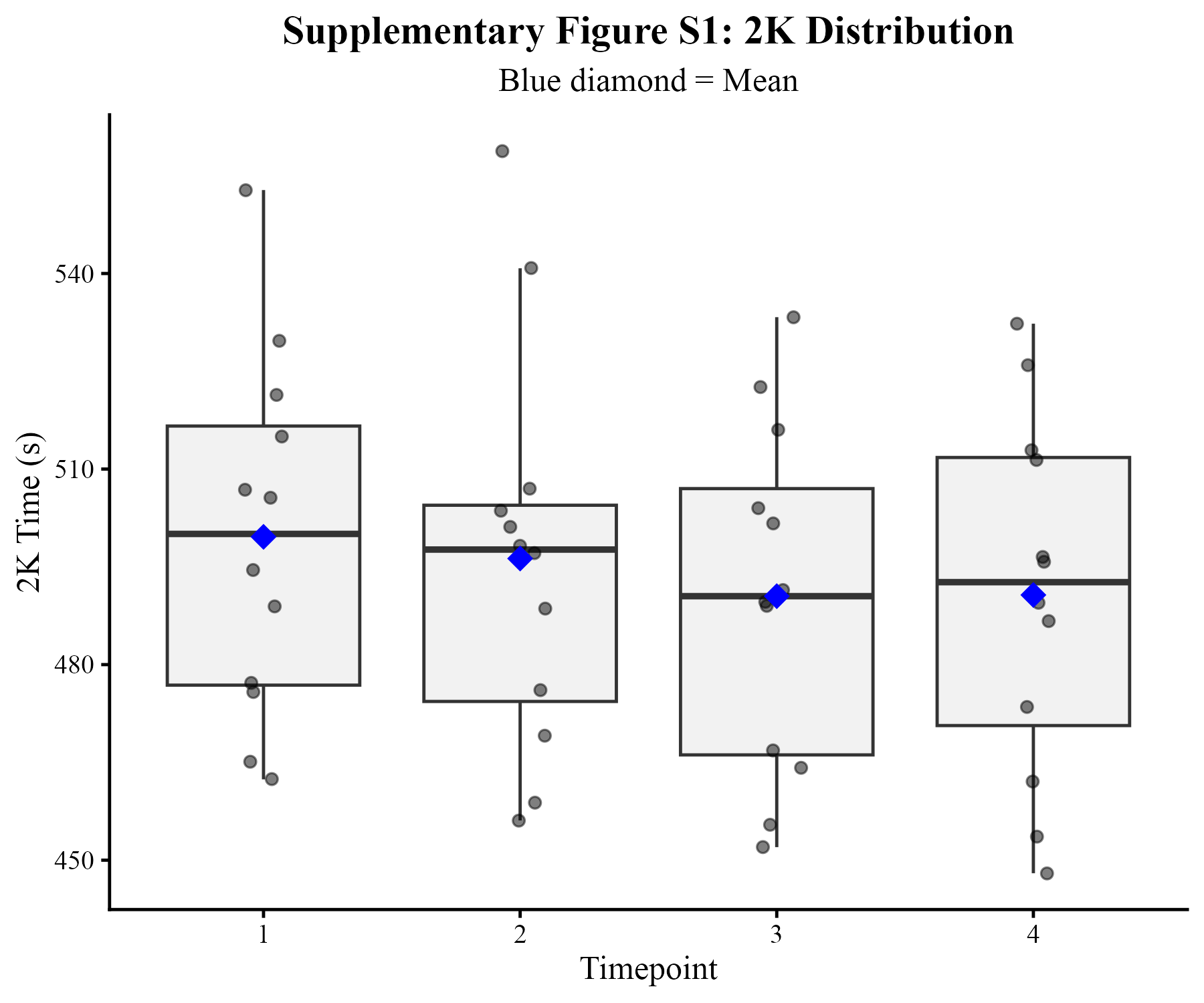

Supplement: Supplementary file 1 [file sports-14-00282-s001.zip › sports-4385241-Supplementary Figure S1_Dist_2K.png]

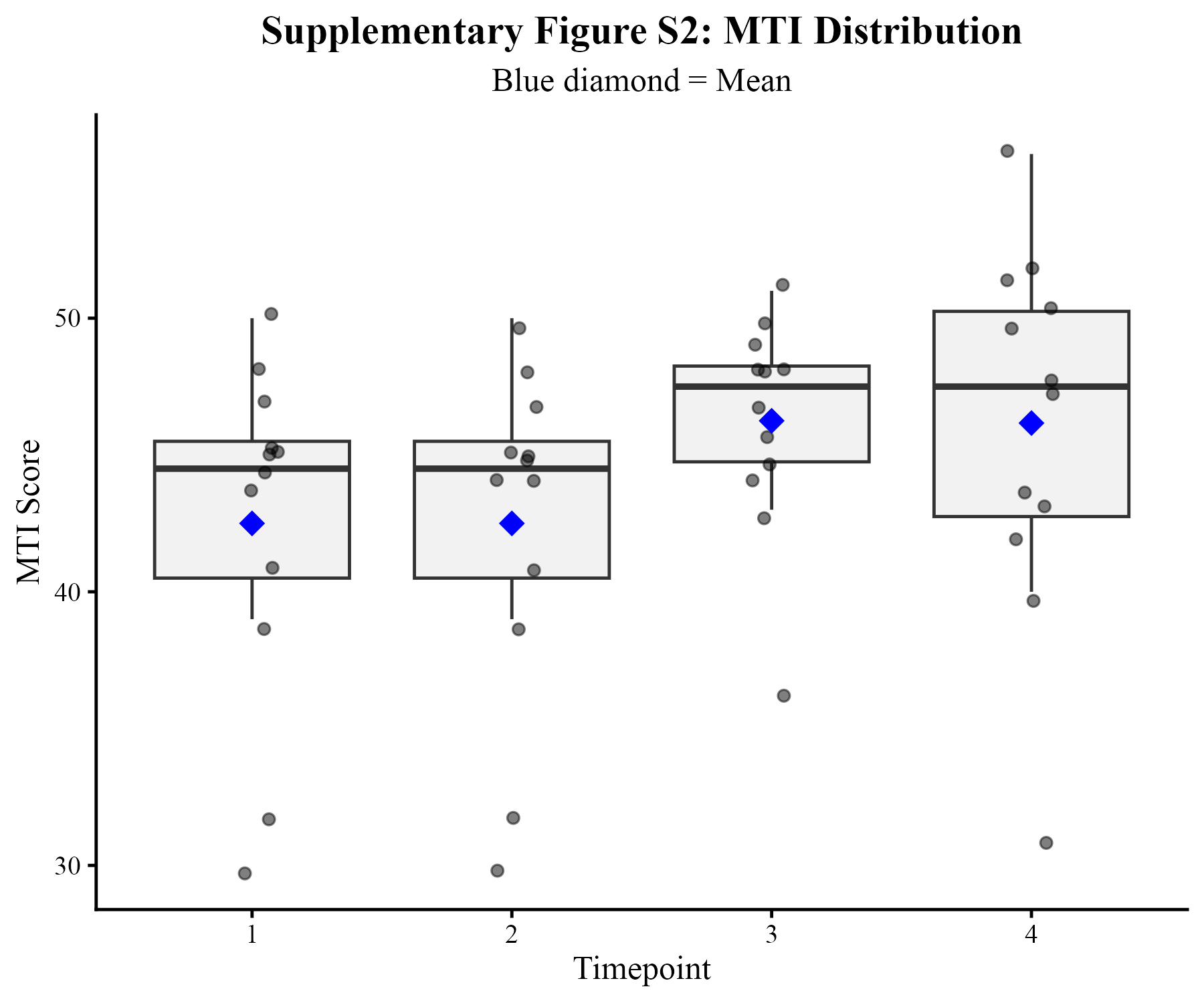

Supplement: Supplementary file 1 [file sports-14-00282-s001.zip › sports-4385241-Supplementary Figure S2_Dist_MTI.png]

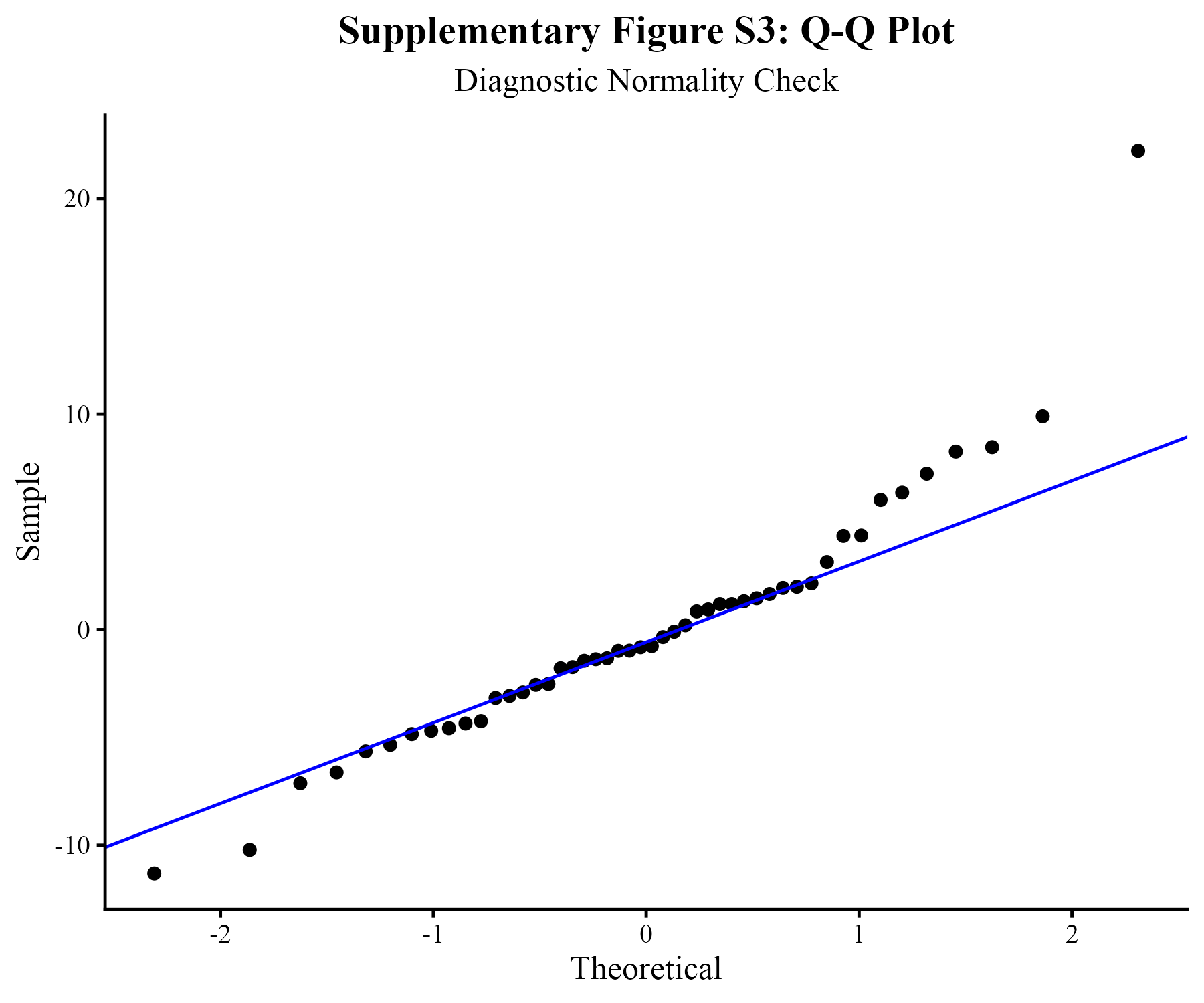

Supplement: Supplementary file 1 [file sports-14-00282-s001.zip › sports-4385241-Supplementary Figure S3_QQ.png]
